# Whole Cell Recognition of *Staphylococcus aureus* Using Biomimetic SPR Sensors

**DOI:** 10.3390/bios11050140

**Published:** 2021-04-29

**Authors:** Neslihan Idil, Monireh Bakhshpour, Işık Perçin, Bo Mattiasson

**Affiliations:** 1 Department of Biology, Hacettepe University, 06800 Ankara, Turkey; nsurucu@hacettepe.edu.tr (N.I.); ipercin@hacettepe.edu.tr (I.P.); 2 Department of Chemistry, Hacettepe University, 06800 Ankara, Turkey; b.monir@hacettepe.edu.tr; 3 Division of Biotechnology, Lund University, 22100 Lund, Sweden; 4 Indienz AB, Annebergs Gård, 26873 Billeberga, Sweden

**Keywords:** SPR biosensor, *Staphylococcus aureus*, micro-contact imprinting, *N*-methacryloyl-L-histidine methyl ester

## Abstract

Over the past few decades, a significant increase in multi-drug-resistant pathogenic microorganisms has been of great concern and directed the research subject to the challenges that the distribution of resistance genes represent. Globally, high levels of multi-drug resistance represent a significant health threat and there is a growing requirement of rapid, accurate, real-time detection which plays a key role in tracking of measures for the infections caused by these bacterial strains. It is also important to reduce transfer of resistance genes to new organisms. The, World Health Organization has informed that millions of deaths have been reported each year recently. To detect the resistant organisms traditional detection approaches face limitations, therefore, newly developed technologies are needed that are suitable to be used in large-scale applications. In the present study, the aim was to design a surface plasmon resonance (SPR) sensor with micro-contact imprinted sensor chips for the detection of *Staphylococcus aureus*. Whole cell imprinting was performed by *N*-methacryloyl-L-histidine methyl ester (MAH) under UV polymerization. Sensing experiments were done within a concentration range of 1.0 × 10^2^–2.0 × 10^5^ CFU/mL. The recognition of *S. aureus* was accomplished by the involvement of microcontact imprinting and optical sensor technology with a detection limit of 1.5 × 10^3^ CFU/mL. Selectivity of the generated sensor was evaluated through injections of competing bacterial strains. The responses for the different strains were compared to that of *S. aureus.* Besides, real experiments were performed with milk samples spiked with *S. aureus* and it was demonstrated that the prepared sensor platform was applicable for real samples.

## 1. Introduction

*Staphylococcus aureus* (*S. aureus*) is one of the most important pathogenic strains causing both hospital-acquired and community-acquired infections that are difficult to treat due to the multi-drug resistance [1,2]. It is necessary to detect this causative microorganism quickly and reliably in order to treat the infections effectively. In particular, food products and natural sources are complex media and therefore, the concentration of the target microorganisms is generally very low. From this point of view, rapid, sensitive, selective, and low-cost detection of these agents is of great importance [3]. Besides, the increased frequency of *S. aureus* with severe infections and even deaths strengthen the requirement of rapid, accurate, and early detection clear [4].

Traditional methods applied to detect these strains have some limitations such as laborious and time consuming procedures in cultivation and identification, besides, the requirement of some test kits [4]. In recent years, several advances have been reported in the literature relying on sensor-based strategies which have beneficial properties applicable in the development of rapid, accurate and sensitive systems [5,6]. Surface plasmon resonance (SPR) based sensors have been introduced to be good candidates for analysis for detection of microorganism and quantification [7]. Real-time, rapid, sensitive, specific, cost-effective, and label-free detection of microorganisms could be possible in favor of these sensors [8]. SPR sensors have predominantly been applied for the detection of *Campylobacter jejuni* [9], *Helicobacter pylori* [10], *Salmonella typhimurium* [11], *Salmonella paratyphi* [12], *Legionella pneumophila* [13], *E. coli O57:H7* [14,15], *Yersinia enterocolitica* [16], *Pseudomonas aeruginosa* [17], *Staphylococcus aureus* [18] and *Vibrio cholerae O1* [19] which are the causes of serious infections including acute bacterial gastroenteritis, peptic ulcer, intestinal tract infections, paratyphoid, lung infections, enterohemorrhagic disease, intestinal and extraintestinal diseases, wound infections, local and systemic difficult-to-treat infections.

**Figure 1:**
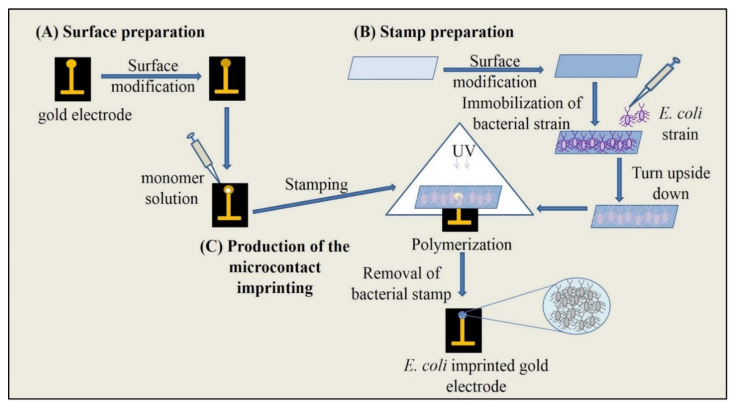
Schematic representation of microcontact imprinting of *E. coli* onto the polymer modified surfaces. (**A**) preparation of electrode surface, (**B**) preparation of bacteria stamps, (**C**) production of the microcontact imprinting (reproduced with permission from [24]).

The effectiveness of SPR-based sensors could be increased by using some biomolecular interactions [6]. Sensors with molecularly imprinted polymers (MIPs) have been proven to be one of the most successful systems which provide tools to develop suitable and trustful platforms in the detection of microorganisms with high selectivity [20,21,22]. Molecular imprinting is a powerful state-of-the-art technology enabling production of specific recognition cites which have similarity in size, shape and chemical functionality to target molecules including also bacterial cells [23]. Furthermore, microcontact imprinting offers some advantages in the formation of imprinted polymers with the placement of immobilized bacterial cells on the surface of the chip. Therefore, the removal of this bacterial stamp from the surface leaves behind a polymer having recognition cites which are complementary with the target microorganism and provide detection of whole bacterial cells [6,8,23,24]. Figure 1 shows the schematic representation of microcontact imprinting of *E. coli* onto the polymer modified gold electrode surfaces. In this study, the similar methodology was applied reported in our previous study [24], presently microcontact imprinting of *S. aureus* onto the polymer modified SPR sensor chips.

In the past decades, several attempts were made to the production of MIPs for the detection of microorganisms along with many accomplished and inspiring publications. Nowadays, molecular imprinting-based sensors are rapidly emerging as a platform to detect whole cells [20,25,26]. Micro-contact imprinting is an approach for forming a polymerization step in which the fingerprint of the target microorganism produced on the surface recognition sites that are compatible with the target molecule is formed. In the literature, microcontact imprinting was applied for some bacterial strains prepared in the presence of organic or inorganic compounds [22]. In a comprehensive study, *Deinococcus radiodurans*, *Escherichia coli CN13*, *Sphaerotilus natans*, and *Bacillus subtilis* were used as template bacterial strains and they were imprinted on organically modified silica thin films via sol-gel technology [27]. With the paid increasing attention to the detection of microorganisms, *Bacillus cereus* was successfully detected using micro contact imprinted organic polymers [28]. Apart from these studies, *Rhodobacter sphaeroides* was detected via microcontact imprinting performed with poly(ethylene-co-vinyl alcohol) [29]. *Vibrio parahaemolyticus* was imprinted in polydimethylsiloxane films by microcontact imprinting [30].

In the present study, a microcontact imprinted SPR sensor for a nosocomial bacterial strain, *S. aureus* was improved. After characterization studies, real-time sensing experiments for the target microorganism were performed. The selectivity of the sensing system was evaluated by using competing microorganisms. Imprinting efficiency of *S. aureus* micro-contact imprinted SPR sensor was examined. In the last step, the applicability of the sensing platform was tested by real sample experiments using microorganism-spiked as well as diluted samples were studied. Furthermore, reusability of the designed system was evaluated.

## 2. Materials and Methods

### 2.1. Materials

Allyl mercaptan, glutaraldehyde (50%, *w/v*), 3-amino-propyltriethoxysilane (APTES), 2-hydroxyethyl methacrylate (HEMA) and ethylene glycol dimethacrylate (EGDMA) chemicals were purchased from Sigma Chemical Co. (St. Louis, MO, USA). α-α’-azoisobutyronitrile (AIBN) was obtained from Fluka (Buchs, Switzerland). N-methacryloyl L-histidine-methyl ester (MAH) was obtained from Research Group Bioreg (Hacettepe University, Ankara, Turkey). All other chemicals were of analytical grade and provided by Merck A.G. (Darmstadt, Germany).

### 2.2. Bacterial Strains

*Staphylococcus aureus* ATCC 25923, *Salmonella paratyphi* ATCC 9150, *Escherichia coli* ATCC 25922, *Bacillus subtilis* ATCC 23857 strains were included in the presented research. Overnight cultures were prepared via incubating the test microorganisms in 100 mL of Luria-Bertani (LB) broth at 37 °C for 18 h in a shaking incubator adjusted to 150 rpm. Measurement of viable bacterial cell counts were carried out with serial 10-fold dilutions using sterile 10 mM phosphate-buffered saline (PBS) (pH 7.4). Suspensions of each dilution (0.1 mL) were inoculated onto LB Agar plates in triplicate and overnight incubation was performed at 37 °C. The formed bacterial colonies were counted to determine the number of colony-forming units per milliliter (CFU/mL). Then, culture suspensions (1 mL) were centrifuged at 3300× *g* for 15 min at 4 °C to separate a cell pellet part containing of the culture. The supernatant was then removed. After centrifugation, the sedimented cells were suspended in sterile 10 mM PBS buffer (pH 7.4). The washing step included suspension of the cells before they were centrifuged again. The supernatant was removed. This procedure was performed few times and the final cell pellet was resuspended in sterile water.

### 2.3. Microcontact Imprinting

#### 2.3.1. Functionalization of Glass Slides

Glass slides were modified in four main steps. The slides were first rinsed with pure ethyl alcohol for 5 min, then, they were conducted with Piranha solution (3:1, (concentrated H_2_SO_4_)/(30% H_2_O_2_), *v/v*) for 20 min. as a third step, they were treated with 3% APTES in toluene (*v/v*) for 2 h for the introduction of amino groups. The last step included exposure for 2 h of glutaraldehyde (3% *v/v*) in phosphate buffer (pH 7.4) onto glass slides in order to derivatize amino groups. After this treatment, the glass slides were washed with deionized water and then dried with nitrogen gas between each step. After glutaraldehyde modification, slides were washed using phosphate buffer and distilled water, respectively. For the immobilization of *S. aureus* cells, 200 µL of bacterial suspension at the concentration of 0.5 × 10^8^ CFU/mL was applied onto the modified surface of the glass slide and they were left at 25 °C for 18 h. After washing with deionized water and drying with nitrogen gas, glass slides with immobilized *S. aureus* were ready for the preparation of microcontact imprinting on the surfaces of SPR chips.

#### 2.3.2. Surface Modification of SPR Chips

The gold surface of SPR chips (GWC Technologies, Madison, WI, USA) was modified by a described method reported in our previous studies [6,8]. For this purpose, treatment with allyl mercaptan (3.0 M) was performed to the SPR chips which were then kept in a fume hood for 18 h. After rinsing with ethyl alcohol for the removal of excess allyl mercaptan, the modified SPR chips were dried in a vacuum oven (200 mmHg, 25 °C).

#### 2.3.3. Preparation of *S. aureus* Imprinted SPR Chips

Microcontact stamping was implemented as an efficient approach to prepare *S. aureus* imprinted SPR chips. In the first step, pre-polymerization was performed by mixing MAH and Cu(NO_3_)_2_·2.5H_2_O (1:1, molar ratio) for 1 h. This was followed by the addition of HEMA (13 µL) and EGDMA (40 µL) to the MAH-Cu(II) complex. After allowing to mix for 5 min, AIBN was added into the monomer solution. The herein mentioned solution was applied onto the surface of SPR chips. The glass slide equipped with target bacterial strain was contacted with the solution on the SPR chip. Then, UV polymerization (100 W, 365 nm, 20 min) was performed. After the removal of the glass slide, the SPR chip was washed with 10 mM phosphate buffer (pH 7.4) and treated with 10 mg/mL lysozyme solution (in PBS buffer, pH 7.4, 10 mM) for removing bacterial residues. The technology described is an improvement of a microcontact imprinting that we earlier have published [31].

### 2.4. Characterization of SPR Chips

Characterization of the chip surfaces was carried out by a JEM 1200 EX Scanning Electron Microscope (SEM, JEOL, Tokyo, Japan). First, the surfaces of SPR chips were rinsed using distilled water and dried with nitrogen gas. Then the surfaces were coated with Au/Pd. Ellipsometry measurements were done by an auto-nulling imaging ellipsometer (Nanofilm EP3, Goettingen, Germany). A four-zone auto-nulling procedure integrating over a sample area of approximately 200 µm × 200 µm followed by a fitting algorithm has been carried out in order to analyze the SPR surface thickness. Phase models including air, polymeric film, gold, chromium and SF10 glass were used for SPR chips.

*S. aureus* imprinted SPR chips were cleaned using ethanol and distilled water and then dried. AFM assay was carried out in METU Central Laboratory for the characterization of three-dimensional imaging of the SPR chip surface.

Water contact angle experiments based on determining the wettability of the chip surface were performed with KRUSS DSA100 (Hamburg, Germany). The hydrophilicity and hydrophobicity of the surfaces of the imprinted and non-imprinted SPR chips were obtained with water contact angle measurements.

### 2.5. Real-Time Detection of S. aureus

Real-time detection of *S. aureus* was carried out with bacterial suspensions at different concentrations via the SPR imager II system (GWC Technologies). After assay the sensor was rinsed. Ethyl alcohol (50%, *v/v*) and lysozyme solution (10 mg/mL) was preferred as regeneration agents.

The equilibration of the sensing system was achieved by passing 10 mM PBS through the system at the flow rate of 150 µL/min. When a stable resonance frequency was obtained, bacterial suspensions were added into the system. The reflectance change (∆R%) was recorded until the signal was stable. Following this, regeneration agents were injected into the system, and then 10 mM PBS was applied in order to re-equilibrate the surface of the chip by removing the remaining residual parts and make the system ready for other injection. SPR chips with non-imprinted polymer (NIP) were prepared by the same imprinting methodology using the same chemicals as for MIP chips except for the immobilization of the bacterial cells onto the surface of glass slides. Imprinting efficiency was determined with the comparison of the sensing detection abilities of NIP and MIP chips. The reflectance changes of the proposed sensing system were examined in both target and competing bacterial strains.

### 2.6. Selectivity of the Sensing

Selectivity of the sensing has a great importance to reveal well-established cell recognition cavities. *B. subtilis*, *S. paratyphi* and *E. coli* strains were used as interfering bacterial strains for the evaluation of the proposed sensing system’s selectivity. Each of the competing bacterial strain samples were maintained constant at a concentration of 10^6^ CFU/mL and cell suspensions were injected to the sensing platform to verify the feasibility of the approach against the corresponding template microorganism. On the other hand, NIP-chips were also functionalized in the same procedure as explained to eliminate the non-selective recognition.

### 2.7. Applicability and Reusability Testing

Analysis of the real samples has a crucial role for the validation along with the evaluation of the applicability of the sensing system. Real sample experiments were performed by using samples of apple juice spiked with bacterial strains that were of interested for the selectivity studies interested. For this purpose, *S. aureus* was spiked at different concentrations (1.0 × 10^2^, 1.0 × 10^4^, 1.0 × 10^5^ CFU/mL) to 10 times diluted samples with PBS (pH 7.4). The analytical cycle contained equilibration-injection-regeneration. The cycles were applied for 5 times. Moreover, the repeatability of the system was investigated by monitoring the reflectivity change during repeated applications performed with the same bacterial concentration.

## 3. Results and Discussion

Pathogenic bacterial strains, including *S. aureus,* carrying resistance genes have become important due to the emergence of permanent treat factors, resistance genes to many fields such as dissemination in the health care settings. Therefore, accurate, rapid, early detection is of great concern and has a crucial role in taking measurements to prevent the spreading of the causative agents of dangerous diseases [32]. Molecular imprinting approach resembles the fundamentals of antibody-based assays which is one of the conventional identification techniques of microorganism [33]. Antibodies have been attractive alternatives to effectively identify a particular microorganism, however there are several challenges still existing such as costs, time consuming and tedious analyzing procedures. On the other hand, natural antibodies are unstable at extreme conditions (pH, temperature etc.). Antigen-antibody interactions are evolved from the basis of molecular recognition. The origin of this recognition mechanism appears as appropriate functioning in three-dimensional interaction. In this respect, multivalent interactions emerged by multifunctional residues that are vital tools to contribute high affinity between the target molecule and its ligand. As a result of these interactions, unique specificity could be obtained by the ability of size, shape, and functional complementarity of antibodies [23]. The resistance genes may be transferred to other organisms, thereby strengthen the threat to the healthcare. Resistance genes can be monitored with high selectivity [34].

MIPs are effective options focusing on various imprinting strategies in literature [35,36,37] for their natural counterparts [38]. There are many promising publications for the selective and specific recognition of microorganisms In this study, microcontact imprinting was performed using histidine-based recognition monomer (MAH) which has the ability to bind with Cu(II) ions. Resultant MAH-Cu(II) complexes enables specific recognition by functionalizing the polymer and provide selectivity with the formation of recognition cavities to the corresponding target molecule. Natural antibodies as recognition receptors are capable of detecting some bacterial cell wall components. By a similar approach, MIPs prepared by template-assisted synthesis assay can be reasonably defined as artificial antibodies containing selective synthetic recognition sites similar to those of biological receptors. The charge heterogeneities have a vital role in recognition, therefore, charge distribution of the imprinted sites allows bacterial capturing with the property of charged cell surfaces. As a result, electrostatic interactions can easily take place in potential recognition [22,39].

There are several studies focusing on imprinting of small molecules [40], peptides [41] and proteins [42,43], however, there is still a great challenge about imprinting of microorganisms [22]. The three-dimensional shape, size and complex surface chemistry of the microorganisms contribute to reduce the sensitivity when using SPR for quantifying microorganisms. Restricted diffusion depth of the electromagnetic field stands for low refractive index. It means that the accessibility of determinants on the bacterial cell wall surfaces hampered interaction with the recognition elements in hydrodynamic conditions in the sensing platform of SPR. Furthermore, heterogenic binding may occur while capturing the microorganisms via imprinted regions. Another point has to be indicated that the shape of microorganisms can not be preserved in some cases at the imprinting step, therefore, there is a growing need for gaining the robustness of assays for microorganisms [32,44].

### 3.1. Characterization of SPR Chips

**Figure 2:**
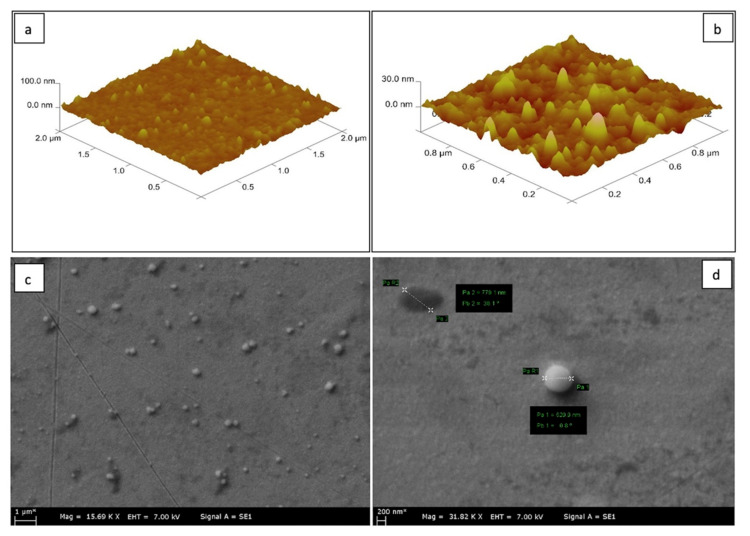
Characterization of *S. aureus*-imprinted SPR sensor chips with AFM and SEM. AFM image of *S. aureus*-imprinted SPR sensor chips, scanning area of 2 µm × 2 µm (**a**), 1 µm × 1 µm (**b**), SEM images of *S. aureus*-imprinted SPR sensor chips (**c**,**d**).

AFM analysis of the *S. aureus*-imprinted SPR chip is given in Figure 2a. A rough image of the surface of the chip was obtained by AFM scanning the area of 2 µm × 2 µm and 1 µm × 1 µm in Figure 2a and Figure 2b, respectively. The images show that the polymerization has taken place successfully. It can be concluded that the thickness of the formed film structure on the chip surface was 50 nm.

SEM analysis of gold surfaces on SPR sensor chip’s and SEM images of *S. aureus* imprinted SPR sensor chip are shown in Figure 2c. The cocci-shape of *S. aureus* clearly appears in Figure 2c. The specific recognition cavity for *S. aureus* formed by the removal of *S. aureus* immobilized glass slides from the surface of the SPR chip can be seen at the left corner of Figure 2d.

Besides, well defined recognition cavities suitable to selectively capture *S. aureus* can be seen in the center of Figure 2d. It is noteworthy to indicate that the diameter of spherical cells of *S. aureus* ranges from 0.5–1.0 μm. Measurements for the cell diameters given in Figure 2d verifies that the target microorganism was successfully imprinted on the chip surface.

**Figure 3:**
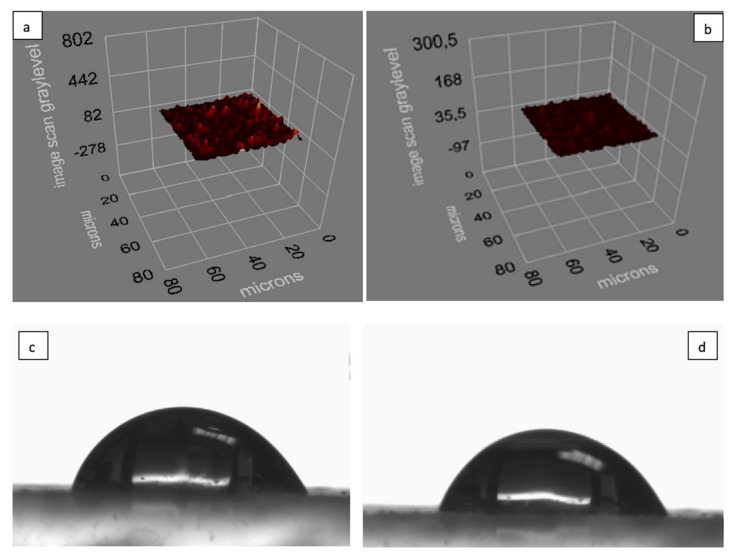
Ellipsometry analysis of *S. aureus-*imprinted SPR sensor chips (**a**) and non-imprinted SPR sensor chips (**b**). Contact angle measurements of *S. aureus*-imprinted SPR sensor chips (**c**) and non-imprinted SPR sensor chips (**d**).

The analysis is showing the thickness surface of micro-contact imprinted and non-imprinted SPR chips as illustrated in Figure 3a and Figure 3b, respectively. The thicknesses of micro-contact imprinted SPR chip was reported as 56.7 ± 0.6, and also, the thicknesses of non-imprinted SPR chip was 33.5 ± 2.3 nm.

The water contact angle measurements of *S. aureus* imprinted and non-imprinted SPR sensor chip were found as 65.8 ± 0.1° (Figure 3c) and 63.4 ± 0° (Figure 3d), respectively. Water contact angles are highly sensitive methods to show the hydrophilicity and hydrophobicity of the chip surface. The value obtained for the bare metallic surface is 0° and this value can increase in presence of a polymeric thin film surface. A hydrophilic surface have an angle smaller than 90° while, the hydrophobicity properties result in an angle larger than 90°. Many polymeric films or nanoparticles show hydrophilic surfaces. The hydrophilic properties of the monomer and cross linker in the *S. aureus* imprinted and non-imprinted thin-film increase the angle value in comparison to that of the bare SPR chip surface. As seen in Figure 3c,d, Cu(II) metal ions coordinated with the MAH functional monomer resulted in hydrophilic properties, the wetting angles of both of the imprinted and non-imprinted chip surfaces were increased.

### 3.2. Real Time Detection of S. aureus

**Figure 4:**
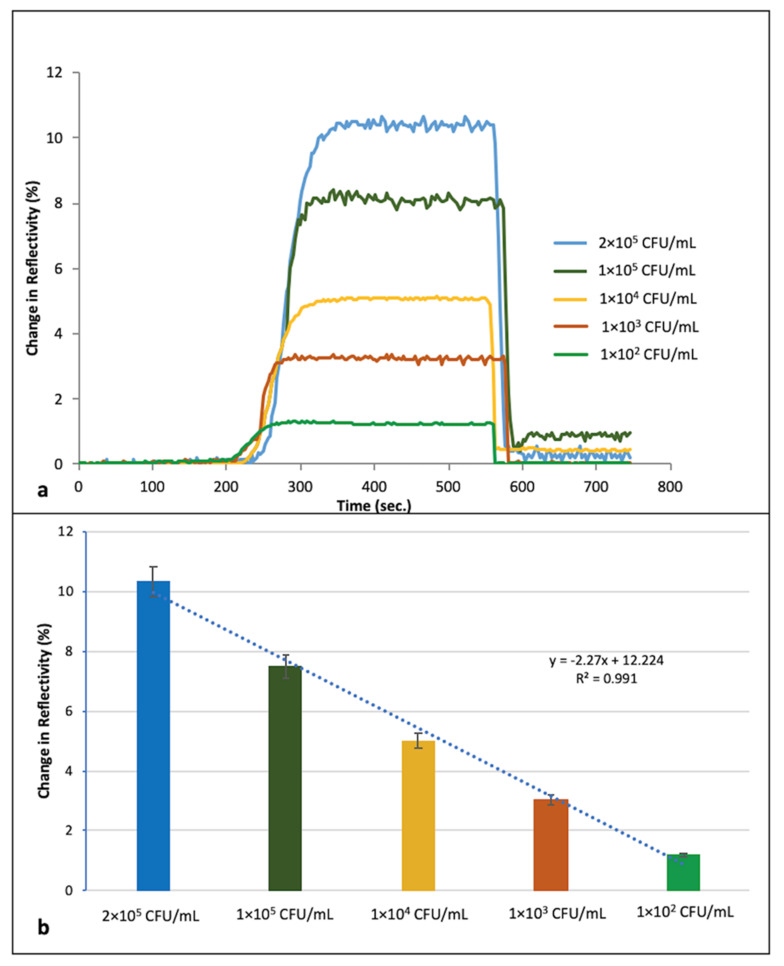
(**a**) Sensorgram of the *S. aureus* imprinted SPR sensor chips (**b**) Calibration curve of *S. aureus* obtained in a range of 1.0 × 10^2^–2.0 × 10^5^ CFU/mL bacterial concentrations.

In Figure 4a, real time responses of *S. aureus*-imprinted SPR sensor chip are shown. The received signals were recorded every minute. The straight baseline was obtained in the first 200 s before injecting *S. aureus* by the effect of running buffer given priority to the system. By the injection of *S. aureus*, it is indicated that target microorganism binds to the recognition sites and a change in reflectivity has started to be recognized. As injection time progresses, the change in reflectivity is increased with the binding of *S. aureus* and the peak height correlates with the amount of cell binding to the surface of SPR chip. Furthermore, increased response from the chip surface was obtained with the increasing concentrations of bacterial cells ranging from 1 × 10^2^–2 × 10^5^ CFU/mL (Figure 4b). The results obtained by the injections before the desorption step demonstrate the efficiency of the generated sensor platform. Capturing cell response of the proposed sensor system has a linear relationship to the bacterial cell concentrations with regression equation of y = −2.27x + 12.224 (R^2^ = 0.991). Limit of detection (LOD) was found to be as 1.5 × 10^3^ CFU/mL.

### 3.3. Selectivity of the Sensing

**Figure 5:**
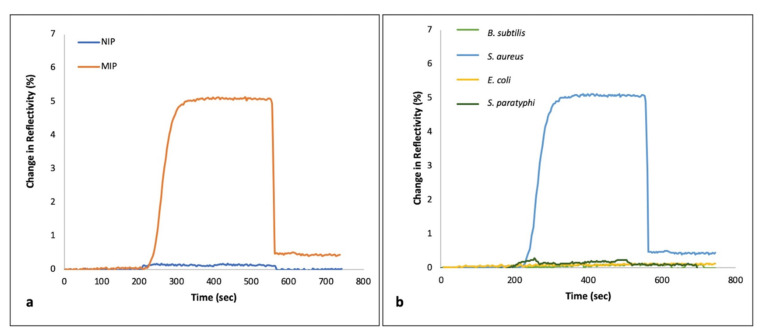
(**a**) Imprinting efficiency of MIP-SPR vs. NIP-SPR sensor chips (**b**) Selectivity of the *S. aureus* imprinted SPR sensor chips against competitor bacterial strains.

In Figure 5a, the sensor responses of MIP and NIP SPR chips obtained from the injection of *S. aureus* were compared with each other. It can be seen that specific recognition cavities for *S. aureus* were successfully formed using MIP sensor chips. In comparison with NIP, both the shape of the microorganism and the surface chemistry of the polymeric structure on the sensor chip makes it possible to monitor high signals. On the contrary, almost no signal was registered from the NIP.

In Figure 5b, the selectivity of the *S. aureus* imprinted sensor chip was indicated by the injection of competitive bacterial strains separately. The highest sensor response was monitored with the injection of *S. aureus* among other competitive bacterial strains (*B. subtilis*, *E. coli* and *S. paratyphi*) due to the shape and chemical function memory of the recognition regions against *S. aureus*. All of the obtained minimal sensor responses against competitors are negligible in comparison to that against *S. aureus*. Selectivity coefficient (*k*) and relative selectivity coefficient (*k’*) of the sensing platform were calculated by following equations as Equation (1) and Equation (2), respectively. Table 1 shows that very low selectivity coefficients were obtained against competitive bacterial strains. In this respect, in the light of these results, the specificity of the sensor has been verified.

*k* = ΔR_target microorganism_/ΔR_competitor_ (1)

*k*’ = *k* _microcontact imprinted_/*k* _non-imprinted_ (2)

### 3.4. Real Sample Experiments and Reusability

**Figure 6:**
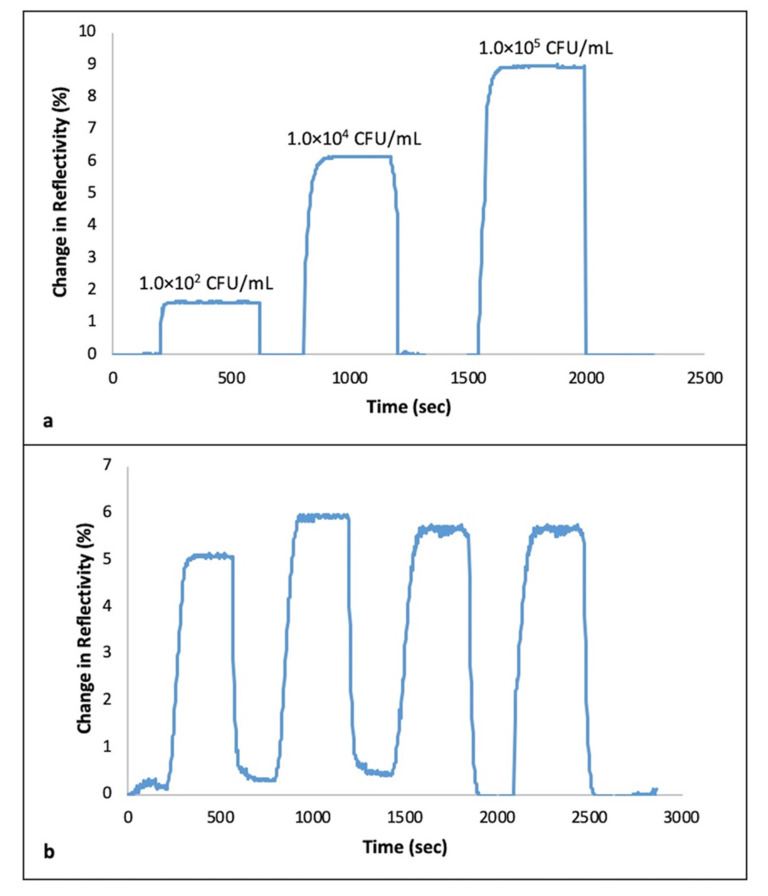
(**a**) Real-time responses of *S. aureus*-imprinted SPR sensor chip showing the sequential injection of *S. aureus*-spiked river milk sample, sample 1: 1.0 × 10^2^ CFU/mL, sample 2: 1.0 × 10^4^ CFU/mL, sample 3: 1.0 × 10^5^ CFU/mL (**b**) Reusability of *S. aureus*-imprinted SPR sensor chip obtained by repeated four equilibration-injection-regeneration cycles.

Milk was preferred to be applied in the real-time experiments due to being an infection resource of *S. aureus*. Defatted cow milk samples were diluted 10 times with the running buffer, then bacterial cell suspensions ranging from 1.0 × 10^2^–1.0 × 10^5^ CFU/mL were injected into the generated sensor system. It was noticed that there was an increase in ΔR value with the increasing concentrations of *S. aureus* in milk, as expected (Figure 6a). Up-to-date studies emphasized that SPR sensing can be applied with complex samples such as blood, urine [45], fruit juices [8], and food with any need to make pre-preparation of the sample.

The reusability of the *S. aureus* imprinted SPR chips was evaluated with the samples spiked with *S. aureus*. Four equilibration–adsorption–regeneration cycles were applied and ΔR% is shown in Figure 6b. The outcome of the result in Figure 6b is quite straightforward that the same SPR could be applied repeatedly performing the appropriate regeneration steps between the processes. Furthermore, it can be concluded that ethanol and lysozyme solution used as regeneration agents are applicable, however the sensing system needs to be reconditioned using running buffer between each new cycle.

## 4. Discussion

Micro-contact imprinting has been notified as one of the most commonly preferred approaches to be applied for whole-cell imprinting of microorganisms. This process with minor corrections could be used to form a sandwich model in which the bacterial cells are situated on the surface of the polymerized structure [22]. In the related literature, there have been some publications using the SPR sensor system for the detection of *S. aureus*. It was emphasized that SPR sensor systems are suitable for early and accurate identification of the organism causing infectious diseases with the requirement of making a fast, real-time, precise, and cost-effective method. In particular, the SPR-DNA array has been designed for the determination of methicillin- resistant *Staphylococcus aureus* (MRSA) strains that cause hospital infection and show multiple antibiotic resistance [46]. In another study, *Staphylococcus aureus* enterotoxin B (SEB), one of the pathogenicity-related toxins of the corresponding bacterial strain in question, was determined at the sub-nanomolar level by SPR system [47].

When the history of *S. aureus* detection with SPR sensors is searched, it can be clearly seen that molecular imprinting technology exploited as effective as antibody and phage-based recognition attempts. There have been some representative studies to clarify this condition. In two different studies performed with antibody based SPR for *S. aureus* detection, LOD value was determined as 10^5^ [18] and 10^6^ [48] CFU/mL, respectively. Similar to this event, *S. aureus* could be detected by lytic phage-based SPR with the LOD value of 10^4^ CFU/mL [49].

In another study which was conducted for the detection of pathogenic microorganisms, *S. aureus* was detected at a concentration of 10^3^ CFU/mL with SPR sensor system using bacteriophage with high specificity. In our study, the same microorganism could be detected with no requirement of a specific molecule along with the approximately same LOD value [50].

Bezdekova et al. prepared magnetic MIPs using dopamine as monomer to obtain *S. aureus* from food samples such as milk and rice. The interesting part of this study to indicate is that the surface of magnetic particles exploited the imprinted layer. Consequently, LOD was reported as 1 × 10^3^ CFU/mL in milk [51].

It should be stressed that use of MIPs technology offers advantages concerning complexibility, stability, reproducibility, costs etc. have been already well covered in the literature. To produce MIPs is far easier than to raise production of antibodies. The MIPs are stable, can be stored at ambient temperature with retained selectivity. In comparative studies between monoclonal antibodies and MIPs it was clear that the affinity was better at the MIPs, furthermore, stability and possibility of reusing the reagent was in favour of the MIPs. A few references are added which highlights some of these aspects [52,53,54].

In our research group, apart from sensor studies, Protein-A imprinted cryogel beads were prepared. These cryogels could be considered as pioneering materials for capturing *S. aureus*. This approach can be attributed to the interaction of *S. aureus* and Protein A, since Protein A is the surface protein present on the *S. aureus* cell wall which acts as a stable cell surface receptor [55]. Our group’s researches in the field of imprinted sensors for whole bacterial cell detection were all performed for Gram negative bacterial strains such as *E. coli*, *Salmonella paratyphi* and *Enterococcus faecalis*. In the present study, it was aimed to detect whole cell of *S. aureus* selected as a model for Gram positive bacterial strain due to being cause of nosocomial infections using imprinted SPR sensors. The detection mechanism could be attributed to the teichoic acids, negatively charged components of the *S. aureus* cell wall, which provide the interaction with MAH-Cu(II) complex.

## 5. Conclusions

In the present study, it has been pointed out that micro-contact imprinting provides simple surface patterning and could easily come together in the design of SPR sensor systems for the detection of pathogenic bacterial strains. Detection is generally needed for the diagnostics of infections and search the risk factors of pathogenic bacterial strains in contaminated samples or area. A thorough evaluation of the related literature yielded that there has been no study performing whole cell imprinting of *S. aureus* in combination with SPR sensor. It is important to indicate that great importance was given to molecular imprinting and MIPs integrated sensing platforms ensure sensitive and outstanding selective detection opportunities against the target microorganisms. As a result, the proposed imprinting based SPR sensors can serve as potential tools for the detection of *S. aureus* in contaminated food sources or even hospital infections, enabling rapid and appropriate control strategies to be developed.

## Figures and Tables

**Table 1:** Selectivity coefficients of *S. aureus* imprinted and non-imprinted SPR chips, *k*: selectivity coefficient for *S. aureus* versus competing bacterial strains, *k*’: relative selectivity coefficient for *S. aureus*-imprinted SPR chip versus non- imprinted SPR chip.

	Reflectance Change, ΔR	Reflectance Change, ΔR	Selectivity Coefficient, *k*	Selectivity Coefficient, *k*	Relative Selectivity Coefficient, *k*, *k*’
Bacterial strains	Imprinted	Non-imprinted	Imprinted	Non-imprinted	
*S. aureus*	5.08	0.15	-	-	-
*B. subtilis*	0.07	0.11	47.47	1.36	34.81
*E. coli*	0.10	0.10	50.80	1.50	33.86
*S. paratyphi*	0.11	0.10	72.57	1.50	48.38

## Data Availability

Not applicable.
